# Response to comment about article ‘changes in memory and cognition during the SARS-CoV-2 human challenge study’

**DOI:** 10.1016/j.eclinm.2025.103143

**Published:** 2025-03-01

**Authors:** Christopher Chiu, Adam Hampshire

**Affiliations:** a Department of Infectious Disease, Imperial College London, London, UK; b Centre for Neuroimaging Science, King’s College London, UK; c Department of Brain Sciences, Imperial College London, UK

We thank Dr Bompart and colleagues for highlighting the importance of the ethical issues surrounding controlled human infection and, in particular, the clinical study on which this analysis was based. While the ethics of the clinical study were out of the scope of this paper, ethical principles and participant safety were the major priority when designing the research and a number of linked publications have discussed these in detail.

The development of the first SARS-CoV-2 human challenge study was undertaken by a consortium of academic, healthcare, policymaker and industry experts. Development of the model was guided by the WHO ethical framework published in 2020, addressing each of the ethical principles set out therein with maximum transparency through media engagement and open access publication. The scientific justification and assessment of risks and benefits were set out in a commentary before the study began.^1^ Extensive public consultation and engagement occurred that established public and participant support for the research.^2^ The coordinated approach by consortium partners enabled the most appropriate clinical site and participant selection to occur to mitigate risks, with a robust informed consent procedure.^3^ Ultimately, the study protocol that was published contained extensive risk mitigation processes and was reviewed by independent experts as well as a specially-convened research ethics committee that gave its favourable opinion to proceed. Later, the ethical reviewers and WHO underwent a formal process of reflection to identify practical lessons that could be carried forward.^4^

The result has been a series of outputs that we believe have made an important contribution to our knowledge of pathogenesis and immunity in the context of a novel virus, with direct implications for pandemic preparedness.^5^ Follow-up of at least 1 year showed that there were no serious adverse events or any long-term negative effects that noticeably affected the study participants, despite the subclinical changes that have informed mechanistic understanding of the effects of infection. Thus, while we hope the ethical acceptability of such studies will remain an active area of discussion, the ethics surrounding this study were demonstrably not ignored and, in contrast, arguably represent one of the most robust considerations of ethical principles of any clinical study in the literature.

## Contributors

Christopher Chiu—writing-original draft.

Adam Hampshire—review & editing.

## Declaration of interests

Adam Hampshire is founder and director of Future Cognition Ltd and co-founder and co-director of H2 Cognitive Designs Ltd, which develop custom cognitive assessment software and provide online cognitive assessment technology as a service respectively, primarily within the research and healthcare sectors. CC has nothing to declare.
